# Supplementary material for: Levofloxacin prophylaxis and parenteral nutrition have a detrimental effect on intestinal microbial networks in pediatric patients undergoing HSCT
Source: Commun Biol. 2023 Jan 13;6:36. doi: 10.1038/s42003-023-04436-7 (PMC9839701; doi:10.1038/s42003-023-04436-7)
Supplement: Supplementary file 2 — Supplementary_figures_and_tables [file 42003_2023_4436_MOESM2_ESM.pdf]

Levofloxacin prophylaxis and parenteral nutrition have a detrimental effect on intestinal microbial networks in pediatric patients undergoing HSCT

Marco Fabbrini[1,2*], Federica D'Amico[1,2], Davide Leardini[3], Edoardo Muratore[3], Monica Barone[1,2], Tamara Belotti[3], Maria Luisa Forchielli[4,5], Daniele Zama[3,5], Andrea Pession[3,5], Arcangelo Prete[3], Patrizia Brigidi[1], Simone Rampelli[2], Marco Candela[2], Silvia Turroni[2*], Riccardo Masetti[3,5]

[1] Microbiomics Unit, Department of Medical and Surgical Sciences, University of Bologna, 40138 Bologna, Italy.
[2] Unit of Microbiome Science and Biotechnology, Department of Pharmacy and Biotechnology, University of Bologna, 40126 Bologna, Italy.
[3] Pediatric Oncology and Hematology Unit "Lalla Seràgnoli", IRCCS Azienda Ospedaliero-Universitaria di Bologna, 40138 Bologna, Italy.
[4] Health Science and Technologies Interdepartmental Center for Industrial Research (CIRI-SDV), University of Bologna, 40100 Bologna, Italy.
[5] Department of Medical and Surgical Sciences (DIMEC), University of Bologna, 40138 Bologna, Italy.
* correspondence: ST silvia.turroni@unibo.it ; MF m.fabbrini@unibo.it

SUPPLEMENTARY INFORMATION

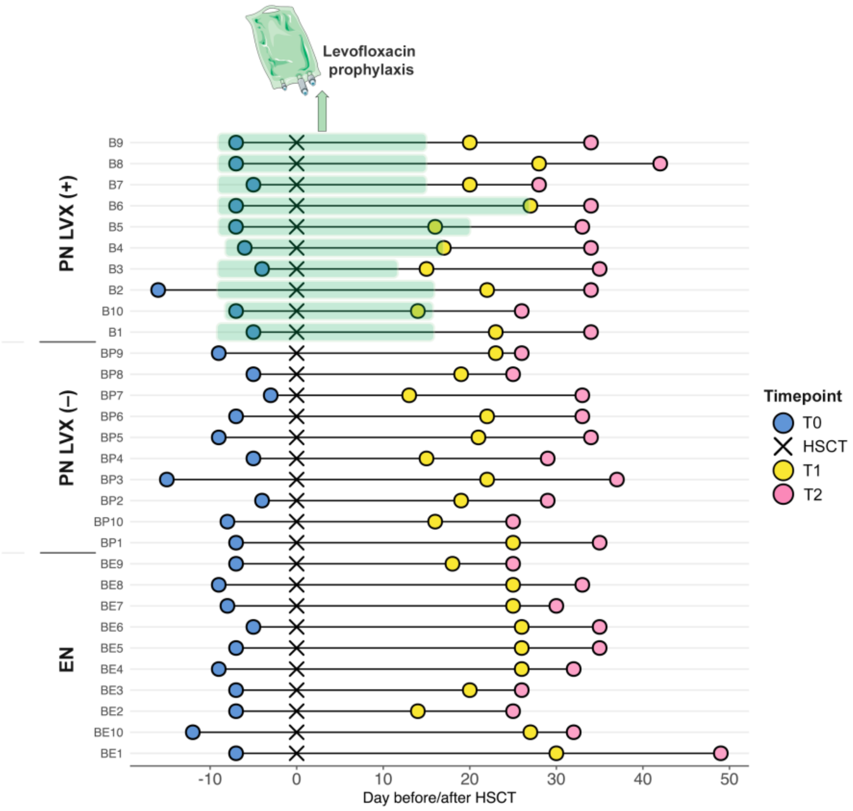

Supplementary Figure 1. Schematic overview of the sampling time for each patient.

Each line represents the time course of an enrolled subject. Distances in timeline are expressed in days with respect to the transplant procedure. Circles are indicative of the sampling time, and the color refers to the timepoint (blue, T0; yellow, T1; pink, T2), while the X indicates the HSCT time (day zero). Patients were stratified by levofloxacin prophylaxis (yes, LVX (+) vs no, LVX (–)) and post-HSCT nutritional support (enteral, EN vs parenteral, PN). The teal box represents the LVX prophylaxis administration window for each PN LVX (+) patient.

The pouch Figure was obtained from Servier Medical Art templates, provided by Servier, licensed under a Creative Commons Attribution 3.0 unported license.

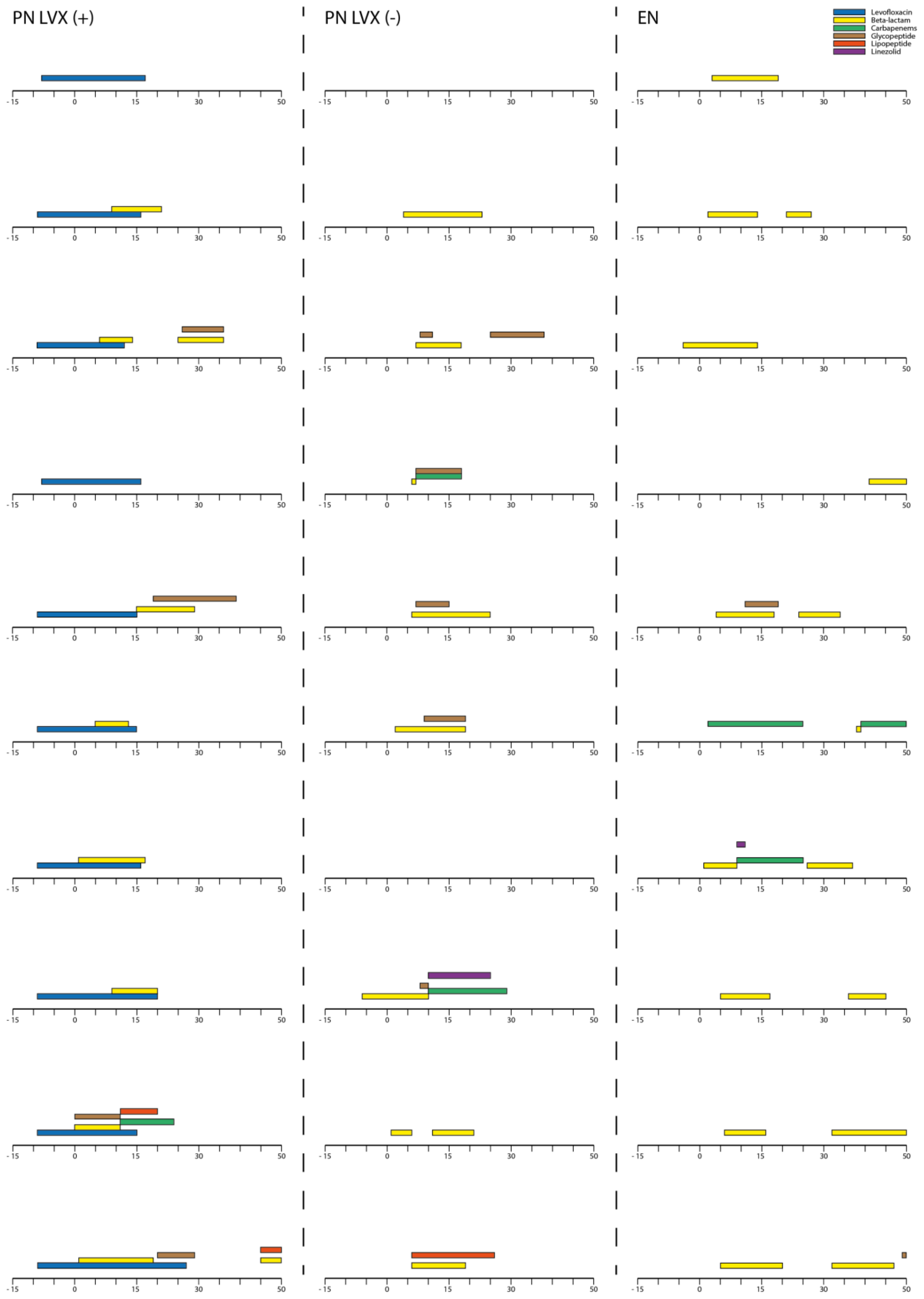

Supplementary Figure 2. Temporal representation of the interim administered antibiotic classes.

For each patient in each group (PN LVX (+), PN LVX (–) and EN), the class of antibiotics administered, and the duration of administration are reported on the timeline.

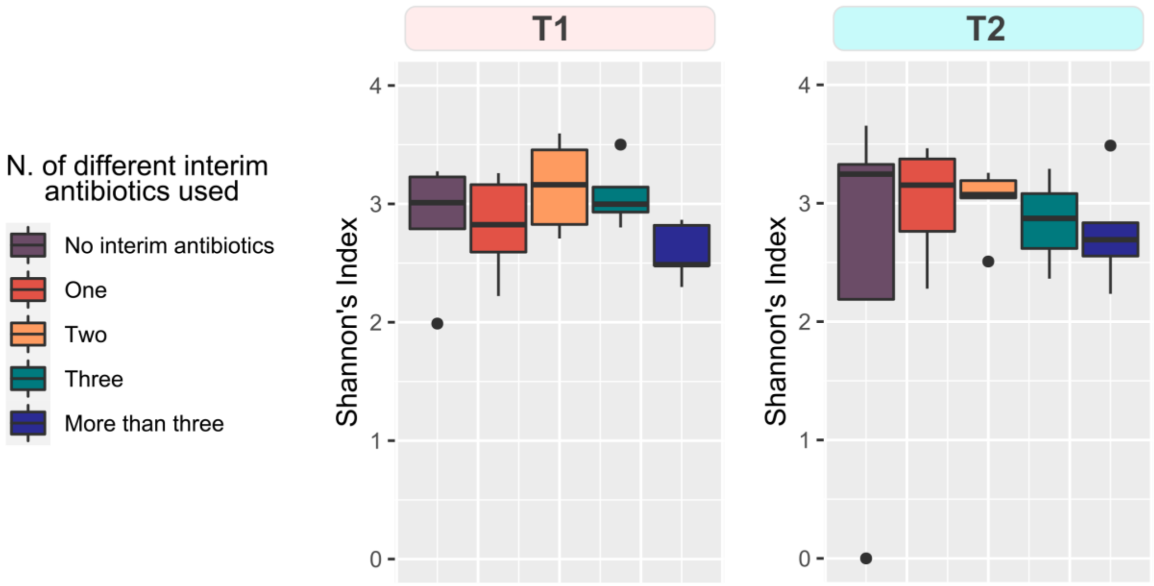

Supplementary Figure 3. Impact of interim antibiotics on gut microbiota alpha diversity. Boxplots showing the distribution of alpha diversity values estimated with the Shannon index at T1 and T2 in patients stratified by the number of different interim non-quinolone antibiotics administered between the date of transplant and the sampled timepoints (see the color legend). No significant differences were found (Kruskal-Wallis test, $p > 0.05$). Whiskers represent range between the first quartile (Q1) and the third quartile (Q3). Data points outside the boundary of the whiskers are plotted as outliers. $n = 30$ at each timepoint; each class based on N. of different interim antibiotics contains at least $n \geq 3$.

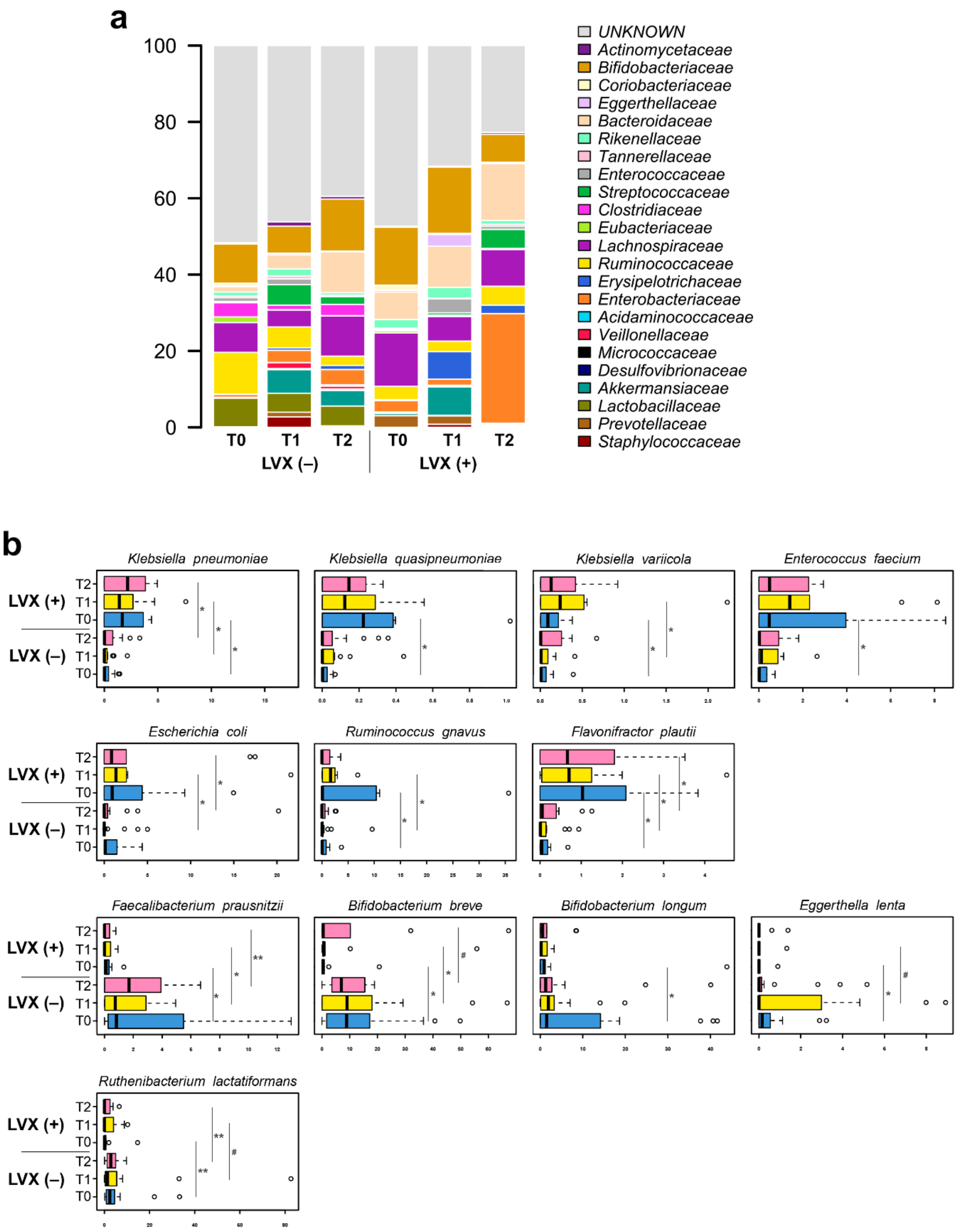

Supplementary Figure 4. Composition of the gut microbiota in relation to levofloxacin prophylaxis.

For this analysis, the EN and PN LVX (–) groups were merged and treated as the LVX (–) group. At baseline (T0), none of the groups received nutritional support and the LVX (+) group already experienced at least 24 hours of fluoroquinolone exposure. (**a**) Family-level composition of the gut microbiota according to LVX prophylaxis. (**b**) Significant differences at species level according to pairwise Wilcoxon rank sum tests with Benjamini-Hochberg correction for multiple comparisons. #, $p < 0.06$ was considered as a trend; *, $p < 0.05$; ** $p < 0.01$. In boxplots, whiskers represent range between the first quartile (Q1) and the third quartile (Q3). Data points outside the boundary of the whiskers are plotted as outliers. $n = 20$ for each LVX (–) timepoint; $n = 10$ for each LVX(+) timepoint.

Supplementary Table 1. Main clinical features of enrolled patients.

Characteristic	Overall	PN LVX (+)	PN LVX (–)	EN
**no.**	30	10	10	10
**Age at HSCT – yr (range)**	10.4 (0.9-19.0)	9.8 (1.0-19.0)	11.1 (0.9-18.1)	10.3 (1.8-17.7)
**Female - no.**	10	2	2	6
**Malignant disease – no.**	22	7	7	8
**Donor – no.**				
MUD	16	3	7	6
MMUD	0	0	0	0
Haplo	6	1	1	4
MSD	8	6	2	0
**Graft type – no.**				
BM	28	10	9	9
PBSC	2	0	1	1
**Intensity of conditioning regimen – no.**				
MAC	30	10	10	10
RIC	0	0	0	0
**Anti-thymocyte globulin treatment – no.**	**16**	**4**	**7**	**5**
**Acute GvHD**				
Grade 0-I	16	4	7	5
Grade II-IV	14	6	3	5
**Bloodstream infections – no.**	**10**	**3**	**7**	**0**

BM, bone marrow; EN, enteral nutrition; GvHD, graft-versus-host disease; Haplo, haploidentical donor; HSCT, hematopoietic stem cell transplantation; LVX (+/–), levofloxacin prophylaxis; MAC, myeloablative conditioning; MMUD, mismatched unrelated donor; MSD, matched sibling donor; MUD, matched unrelated donor; PBSC, peripheral blood stem cells; PN, parenteral nutrition; RIC, reduced-intensity conditioning.

Supplementary Table 2. Information regarding bloodstream infections with positive blood cultures. Patient BP7 experienced three different infections with positive blood culture.

Patient code	Patient group	Pathogen	Clinical findings	Days of positive blood culture	Resistance to quinolones	Resistance to beta-lactams
BP2	PN LVX (-)	*Staphylococcus aureus*	Fever in neutropenia	7	-	-
BP13	PN LVX (-)	*Enterococcus faecalis, Staphylococcus epidermidis*	Fever in neutropenia	6	-	-
BP6	PN LVX (-)	*Escherichia coli*	Fever in neutropenia	2	-	+
BP8	PN LVX (-)	*Staphylococcus aureus*	Pneumonitis	94	-	-
BP7	PN LVX (-)	*Klebsiella pneumoniae*	Fever in neutropenia	6	-	+
BP7	PN LVX (-)	*Klebsiella pneumoniae*	Fever in neutropenia	8	+	+
BP7	PN LVX (-)	*Pseudomonas aeruginosa*	Fever in neutropenia	12	-	-
BP5	PN LVX (-)	*Enterococcus faecalis, Streptococcus mitis*	Fever	57	-	-
BP1	PN LVX (-)	*Staphylococcus epidermidis*	Fever in neutropenia	5	-	+
B8	PN LVX (+)	*Rothia mucilaginosa*	Fever in neutropenia	15	-	-
B9	PN LVX (+)	*Staphylococcus epidermidis*	Fever in neutropenia	0	+	+
B6	PN LVX (+)	*Staphylococcus haemolyticus*	Fever in neutropenia	20	+	+

EN, enteral nutrition; LVX, levofloxacin prophylaxis; PN, parenteral nutrition
