# Supplementary material for: Levofloxacin prophylaxis and parenteral nutrition have a detrimental effect on intestinal microbial networks in pediatric patients undergoing HSCT
Source: Commun Biol. 2023 Jan 13;6:36. doi: 10.1038/s42003-023-04436-7 (PMC9839701; doi:10.1038/s42003-023-04436-7)
Supplement: Supplementary file 3 — Reporting Summary [file 42003_2023_4436_MOESM3_ESM.pdf]

nature portfolio

Corresponding author(s): Marco Fabbrini

Last updated by author(s): 04 January 2023

Reporting Summary

Nature Portfolio wishes to improve the reproducibility of the work that we publish. This form provides structure for consistency and transparency in reporting. For further information on Nature Portfolio policies, see our Editorial Policies and the Editorial Policy Checklist.

Statistics

For all statistical analyses, confirm that the following items are present in the figure legend, table legend, main text, or Methods section.

n/a	Confirmed	
☐	☒	The exact sample size (*n*) for each experimental group/condition, given as a discrete number and unit of measurement
☐	☒	A statement on whether measurements were taken from distinct samples or whether the same sample was measured repeatedly
☐	☒	The statistical test(s) used AND whether they are one- or two-sided<br>*Only common tests should be described solely by name; describe more complex techniques in the Methods section.*
☐	☒	A description of all covariates tested
☐	☒	A description of any assumptions or corrections, such as tests of normality and adjustment for multiple comparisons
☐	☒	A full description of the statistical parameters including central tendency (e.g. means) or other basic estimates (e.g. regression coefficient) AND variation (e.g. standard deviation) or associated estimates of uncertainty (e.g. confidence intervals)
☐	☒	For null hypothesis testing, the test statistic (e.g. *F*, *t*, *r*) with confidence intervals, effect sizes, degrees of freedom and *P* value noted<br>*Give P values as exact values whenever suitable.*
☒	☐	For Bayesian analysis, information on the choice of priors and Markov chain Monte Carlo settings
☒	☐	For hierarchical and complex designs, identification of the appropriate level for tests and full reporting of outcomes
☒	☐	Estimates of effect sizes (e.g. Cohen's *d*, Pearson's *r*), indicating how they were calculated

Our web collection on statistics for biologists contains articles on many of the points above.

Software and code

Policy information about availability of computer code

Data collection	No software was used for data collection.
Data analysis	Statistical analyses were performed in R 4.2.2. Networks were computed with the igraph Rpackage. Statistical tests were performed using functions from the stats base Rpackage. The software Cytoscapes was used for plotting the networks. Compositional details from shotgun metagenomics data were obtained using the MetaPhlAn3 software and functional insights were obtained with the HuMaNn3 pipeline

For manuscripts utilizing custom algorithms or software that are central to the research but not yet described in published literature, software must be made available to editors and reviewers. We strongly encourage code deposition in a community repository (e.g. GitHub). See the Nature Portfolio guidelines for submitting code & software for further information.

Data

Policy information about availability of data

All manuscripts must include a data availability statement. This statement should provide the following information, where applicable:

- Accession codes, unique identifiers, or web links for publicly available datasets
- A description of any restrictions on data availability
- For clinical datasets or third party data, please ensure that the statement adheres to our policy

High-quality paired-end sequences have been deposited in the National Center for Biotechnology Information Sequence Read Archive (NCBI SRA) under the Project code PRJNA914532. (continues in the following page...)

Numerical source data (raw data) used for graphs and charts is available on Figshare at the following DOIs: Figure 1 - 10.6084/m9.figshare.21749753; Figure 2 - 10.6084/m9.figshare.21749768; Figure 3 - 10.6084/m9.figshare.21749777; Figure 4 - 10.6084/m9.figshare.21749780; Figure 5 - 10.6084/m9.figshare.21749786.

Human research participants

Policy information about studies involving human research participants and Sex and Gender in Research.

Reporting on sex and gender	Gender and sex information were not collected
Population characteristics	Pediatric patients receiving hematopoietic stem cell transplantation (HSCT), with or without intravenous levofloxacin prophylaxis. After transplant some received enteral feeding, whilst others received parenteral feeding. Faeces were collected before the conditioning regimen, at neutrophil engraftment and starting from 25 days post-HSCT.
Recruitment	Eligible patients were selected from previous studies (Biagi et al., 2019 and D'Amico et al., 2019) and matched for HSCT characteristics.
Ethics oversight	Ethics Committee of Sant'Orsola-Malpighi University Hospital

Note that full information on the approval of the study protocol must also be provided in the manuscript.

Field-specific reporting

Please select the one below that is the best fit for your research. If you are not sure, read the appropriate sections before making your selection.

☒ Life sciences ☐ Behavioural & social sciences ☐ Ecological, evolutionary & environmental sciences

For a reference copy of the document with all sections, see nature.com/documents/nr-reporting-summary-flat.pdf

Life sciences study design

All studies must disclose on these points even when the disclosure is negative.

Sample size	No sample-size calculation was performed, all possible patients matching the inclusion criteria who gave informed consent were included
Data exclusions	No data were excluded from the analysis
Replication	Multiple patients were considered for each category (enteral nutrition vs parenteral nutrition vs levofloxacin prophylaxis followed by parenteral nutrition) at each timepoint
Randomization	Allocation was not random, but dictated by the therapy each patient underwent during hospitalization
Blinding	Investigators were not blinded to allocation (see above)

Reporting for specific materials, systems and methods

We require information from authors about some types of materials, experimental systems and methods used in many studies. Here, indicate whether each material, system or method listed is relevant to your study. If you are not sure if a list item applies to your research, read the appropriate section before selecting a response.

Materials & experimental systems

n/a	Involved in the study	
☒	☐	Antibodies
☒	☐	Eukaryotic cell lines
☒	☐	Palaeontology and archaeology
☒	☐	Animals and other organisms
☒	☐	Clinical data
☒	☐	Dual use research of concern

Methods

n/a	Involved in the study	
☒	☐	ChIP-seq
☒	☐	Flow cytometry
☒	☐	MRI-based neuroimaging
